# Zynteglo: Betibeglogene autotemcel – An innovative therapy for β- thalassemia patients

**DOI:** 10.1016/j.amsu.2022.104624

**Published:** 2022-09-13

**Authors:** Adam Ali Asghar, Yumna Khabir, Mahnoor Rehan Hashmi

**Affiliations:** Department of Medicine, Dow University of Health Sciences, Mission Rd, New Labour Colony Nanakwara, Karachi, Sindh, Pakistan

β-Thalassemia is a heterogeneous autosomal recessive hereditary anemia characterized by reduced or absent β-globin chain synthesis [1]. It is highly prevalent with 80–90 million reported cases (1.5% of the global population) [1]. β-Thalassemia is usually due to gene mutations (point mutations in promoter or splicing sites). Two β genes are present on chromosome 11; mutations result in absent (β0) or diminished (β+) production of the β-globin chain [1]. It is seen frequently in individuals of Southeastern and Southern Asia, African and Mediterranean descent [1]. There are three clinical and hematological conditions - Thalassemia major, Thalassemia intermedia, and Thalassemia minor and severe transfusion-dependent anemia—recognized each with varying degrees of severity [1].

β-Thalassemia major is the most severe form of the disease and presents with severe anemia shortly after birth; high HbF at birth provides temporary protection [1]. Due to the α-globin chain's continued production and absence of β-Globin, α-Globin tetramers accumulate and precipitate in the erythroid precursors forming inclusion bodies that, bound to the membrane skeleton, cause oxidative membrane damage and extensive premature destruction by apoptosis of the RBC precursors in the bone marrow (ineffective erythropoiesis) [1]. Hemolysis plays a secondary role. Abnormal growth of erythroid marrow in the medullary and extramedullary sites leads to characteristic facial and skull deformities. Cortical thinning and pathological fractures of long bones may result as well as extramedullary erythropoietin tissue masses [1]. Chronic transfusions are often necessary, but they are associated with secondary hemochromatosis [2]. β-Thalassemia minor and β-Thalassemia intermedia are milder forms of the disease and do not present with severe problems.

The treatment options available for β-Thalassemia major are limited and associated with a wide range of complications. The frequently suggested therapy to maintain healthy levels of hemoglobin (Hb) is blood transfusion. The maximum range of RBCs that can be transfused without giving a spike increase in volume of blood is 15–20 ml/kg [3]. The regular transmission of blood results in accumulation of iron within the reticuloendothelial system as the iron excretory mechanisms of the body are compromised [3]. The deposited iron harms endocrine glands, blood vessels, enhances the risk of liver carcinoma, cirrhosis and can lead to diabetes and infertility [3]. Prolong blood transfusion products may also pave the way for pathologic hepatitis [3]. Also finding a compatible blood donor is a barrier itself [3]. The other solutions include bone marrow transplant, iron chelation therapy and splenectomy [3].

Considering the issues previous strategies present with, approval of a one-time cell-based gene therapy (Zynteglo: betibeglogene autotemcel or beti-cel) by Food and Drug Administration (FDA) on 17h August 2022 is a big achievement in the scientific world [4]. Betibeglogene autotemcel is being assessed as gene therapy in patients with transfusion-dependent β-thalassemia. It is a potential curative treatment to correct the globin chain imbalance, thus potentially improving production of normal hemoglobin, erythropoiesis and chronic anemia [5]. Beti-cel adds functional copies of a modified HBB gene having an amino acid substitution (T→Q) at position 87 and also adds β-globin regulatory elements in hematopoietic stem cells through CD34^+^ cells having replicative, defective, self-inactivating BB305 lentiviral vector [6]. The patients go through autologous hematopoietic stem cell mobilization and harvesting. The harvested cells are transduced ex vivo with self-inactivating lentiviral vectors which insert a gene construct having globin gene and other elements required for expression [6]. Then these cells are introduced into the patient where they replicate and repopulate the normal blood [6]. A recent open-label study in 2022, evaluated efficiency and safety of beti-cel in 23 patients of transfusion dependent β thalassemia out of which 20 demonstrated transfusion independence [6]. Average hemoglobin levels at the time of infusion were noted to be 11.7 g/dL and 8.7 g/dL after 12 months [6]. The most common non-laboratory adverse reactions (≥20%) were mucositis, febrile neutropenia, vomiting, pyrexia, alopecia, epistaxis, abdominal pain, musculoskeletal pain, cough, headache, diarrhea, rash, constipation, nausea, decreased appetite, pigmentation disorder, and pruritus. The most common Grade 3 or 4 laboratory abnormalities (>50%) include neutropenia, thrombocytopenia, leukopenia, anemia, and lymphopenia. The few risks associated with use of beti-cel are delayed platelet engraftment which may cause thrombocytopenia and bleeding, neutrophil engraftment failure (failure to achieve 3 consecutive absolute neutrophil counts on different days), insertional oncogenesis where patients may develop hematologic malignancies and hypersensitivity reactions due to dimethyl sulfoxide (DMSO). Beti-cel should not be administered in patients with anti-retroviral and hydroxyurea use. It is also not recommended to be administered on pregnant and breastfeeding women [7].

## Ethical approval

The approval was not needed.

## Please state any sources of funding for your research

The authors have no financial or proprietary interest in the subject matter of this article.

## Author contribution

Adam Ali Asghar; Conceptualization, literature review and manuscript writing.

Yumna Khabir; Literature review and manuscript writing.

Mahnoor Rehan Hashmi; Literature review and manuscript writing.

## Please state any conflicts of interest

The authors have no conflict of interest.

## Registration of research studies

1. Name of the registry:

2. Unique Identifying number or registration ID:

3. Hyperlink to your specific registration (must be publicly accessible and will be checked):

## Guarantor

Adam Ali Asghar, Yumna Khabir and Mahnoor Rehan Hashmi.

## Consent

No consent was required.
